# Synergism of wt-p53 and synthetic material in local nano-TAE gene therapy of hepatoma: comparison of four systems and the possible mechanism

**DOI:** 10.1186/s12885-019-6162-7

**Published:** 2019-11-20

**Authors:** Gaopeng Li, Wenqin Kang, Mingliang Jin, Lidong Zhang, Jian Zheng, Kai Jia, Jinfeng Ma, Ting Liu, Xueyi Dang, Zhifeng Yan, Zefeng Gao, Jun Xu

**Affiliations:** 1 Department of General Surgery, Shanxi Cancer Hospital, Shanxi Medical University, Taiyuan, Shanxi Province China; 2 Department of Critical Care Medicine, First Hospital of Shanxi Medical University, Taiyuan, Shanxi Province China; 3 Department of Anesthesia, Taiyuan Central Hospital, Taiyuan, Shanxi Province China; 4 Department of General Surgery, Qingxu People’s hospital, Taiyuan, Shanxi Province China; 5 Department of General Surgery, Shanxi Cancer Hospital, Shanxi Medical University, Taiyuan, Shanxi Province China; 6 Department of General Surgery, Shanxi Bethune hospital, Shanxi academy of medical sciences, Taiyuan, Shanxi Province China

**Keywords:** Nanoparticles, Gene transfer techniques, Hepatocellular carcinoma, Combined therapy, Rabbits, VX2 tumor

## Abstract

**Background:**

TAE-gene therapy for hepatoma, incorporating the tumor-targeted therapeutic efficacy of trans-arterial embolization, hydroxyapatite nanoparticles (nHAP) and anti-cancer wild-type p53 gene (wt-p53), was presented in our former studies (Int J Nanomedicine 8:3757-68, 2013, Liver Int 32:998-1007, 2012). However, the incompletely antitumoral effect entails defined guidelines on searching properer materials for this novel therapy.

**Methods:**

Unmodified nHAP, Ca^(2+)^ modified nHAP, poly-lysine modified nHAP and liposome were separately used to form U-nanoplex, Ca-nanoplex, Pll-nanoplex, L-nanoplex respectively with wt-p53 expressing plasmid. The four nanoplexs were then applied in vitro for human normal hepacyte L02 and hepatoma HePG2 cell line, and in vivo for rabbits with hepatic VX2 tumor by injection of nanoplexs/lipiodol emulsion into the hepatic artery in a tumor target manner. The distribution, superficial potential, physical structure, morphology and chemical compositions of nanoplexs were evaluated by TEM, SEM, EDS etc., with the objective of understanding their roles in hepatoma TAE-gene therapy.

**Results:**

In vitro, L-nanoplex managed the highest gene transferring efficiency. Though with the second highest transfection activity, Pll-nanoplex showed the strongest tumor inhibition activity while maintaining safe to the normal hepacyte L02. In fact, only Pll-nanoplex can combine both the antitumoral effect to HePG2 and safe procedure to L02 among the four systems above. In vivo, being the only one with successful gene transference to hepatic VX2 tumor, Pll-nanoplex/lipiodol emulsion can target the tumor more specifically, which may explain its best therapeutic effect and hepatic biologic response. Further physical characterizations of the four nanoplexs suggested particle size and proper electronic organic surface may be crucial for nano-TAE gene therapy.

**Conclusion:**

Pll-nanoplex is the most proper system for the combined therapy due to its selectively retention in liver cancer cells, secondary to its morphological and physico-chemical properties of nanometric particle size, steady emulsion, proper organic and electronic surface.

## Background

Hepatocellular carcinoma (HCC) is among the most common and lethal cancers worldwide, especially in China [3, 4]. To date, the only possible curative treatments are liver resection and transplantation. However, most cases escape the early detection of small HCCs and opportunity for radical resection [5]. In addition, the severely impaired hepatic functional reserve, the occurrence of relapse and the shortage of organs also limited the operations. All the published gene trials on advanced hepatocellular carcinoma patients have been unsuccessful, due to a lack of understanding of hepatocarcinogenesis and tumor progression [6]. So, most patients with unresectable HCC have to resort to various nonoperative strategies [7], among which, combined therapy is the best solution [8]. Wild-type p53 (wt-p53) is a house-keeping tumour suppressor that is frequently mutated and disfunctional in more than 50% of HCCs. Former study [1, 9, 10] successfully combined wt-p53 gene therapy, transcatheter arterial embolization (TAE) and antitumoral nanoparticle for hepatoma by exploiting poly-lysine modified hydroxyapatite nanoparticles (Pll-nHAP) to serve as both embolic material and therapeutic target gene vector at the same time. Unfortunately, ideal transfection activity and completely tumor eradication were not achieved and necessitate further improvements. Moreover, there is no systemic research on identifying the necessary physico-chemical properties of synthetic material for this innovative combined therapy. In this study, we compared the application of Ca^(2+)^ modified nHAP, unmodified nHAP, liposome and the former-utilized Pll-nHAP system in TAE-gene therapy both in vitro and in vivo. From that comparison, we conclude the necessary similarities and propose basic guidelines for selecting synthetic inorganic materials in novel strategy of nano-TAE gene therapy.

## Methods

### Materials

Hydroxyapatite nanoparticles (nHAP), mean radius of 20 nm and zeta potential of − 50.1 mV, were synthesized by improved precipitation method of Biomaterial Center of Wuhan University of Technology (Wuhan, China) [11, 12]. nHAP solution (50 mg nHAP/ 1 ml 0.9% NaCl) is first sterilized by high pressure steam sterilization and then emulsificated by ultrasonic processor (H65025T, USA) for 15 mins (0.6~0.8 mA). Human hepatoma HepG2 (Cat.No.: GDC0024) and normal hepatocyte L02 cell line (Cat.No.: CL0192) were purchased from China Center for Type Culture Collection (CCTCC), and were maintained in DMEM medium supplemented with 10% fetal bovine serum (FBS, Invitrogen, USA.) and kanamycin (100 mg/ml) at 37 °C in 5% CO2 humidified atmosphere. Plasmid DNA (pDNA) PEGFP-C2 and its wt-p53 containing subclone (PEGFP-C2-wt-p53) were prepared and investigated according to our former studies [1, 9, 10]. New zealand rabbits, female or male, weighing 2.5-3.5 kg at approximately 17 to 19 weeks of age, were obtained from the laboratory animal center of Shanxi medical university. VX2 tumor-bearing rabbits were presented by Zhongnan Hospital of Wuhan university. All the animal experiments and breeding were performed under conditions approved by the Ethics Committee of Shanxi medical university, in compliance with the NIH guidelines and items for care and use of laboratory animals and in accordance with the Chinese relevant legislation on animal use. The VX2 models were prepared according to procedures described in the former reports [1, 9, 10]. All the animals were operated under general anesthesia, intramuscular injection of 0.2 ml per kilo body weight Sumianxin (Quartermaster University of PLA, China), by a veterinary anesthetist. The animals for harvesting samples were euthanised by cervical dislocation after ether anesthesia at the completion of the study. The animals for observation of survival date were taken care till the natural death.

### Preparations of different nanoplexs and confirmation of proper charge ratio of nHAP /pDNA

(1): Pll-nHAP and Ca-nHAP were designed and prepared by using 0.3 ml 0.1% Pll or 0.3 ml 0.1% Cacl_2,_ as reported in our former work [10]. (2): For preparation of the nanoplexs, different amount (1, 5, 10, 15, 20, 25, 50 μg) of various nHAP (U-nHAP, Ca-nHAP, Pll-nHAP) or 2.5 μl lipofectamine 2000 (Invitrogen, USA) were mixed and incubated separately with 1 μg pDNA PEGFP-C2 according to the former reports [10, 13] or commercial protocol. (3): Cytotoxicity of various nHAP based nanoplex (including 1 μg/ml pDNA PEGFP-C2 and 1, 5, 10, 15, 20, 25, 50 μg/ml nHAP, Ca-nHAP or Pll-nHAP separately) for HepG2 and L02 were evaluated by MTT to exploit and confirm a proper charge ratio of nHAP /pDNA with maximal HepG2 cytotoxicity and minimal L02 cytotoxicity. The incubation time of the nanoplexs for MTT is 72 h. All the following tests in this study utilized nHAP nanoplexs with that proper charge ratio (w/w nanoparticles: pDNA PEGFP-C2-wt-p53 15:1). Comparative evaluation of the four nanoplexs was carried out through investigating the cell viability, transfections efficiency, necrosis and apoptosis of HepG2 and L02 by MTT, fluorescence microscope (FM) and flowcytometry respectively. pDNA with normal saline solution served as controls. The experiment details are according to our former reports [1, 9, 10]. (4): The nHAP/lipiodol and nanoplex/lipiodol W/O emulsions were prepared by emulsionizing 1 ml lipiodol and 1 ml nanoplexs (containing 3.75 mg various nHAP and 250 μg of pEGFPC2-wt-p53 pDNA), according to the pumping method in our former report, followed by storage at room temperature prior to the surgical procedure [1, 9, 10]..

### Specific gene delivery and retention of nanoplex/lipiodol emulsion to VX2 tumor in vivo

The surgical procedures were taken by selective catheterization to the left hepatic artery of VX2 tumor-bearing rabbits, followed by trans-arterial injection 2 ml of random one emulsion per kg body weight: pDNA/lipiodol (A, 13 animals), L-nanoplex/lipiodol (B, 10 animals), U-nanoplex/lipiodol (C, 10 animals), Ca-nanoplex/lipiodol (D, 10 animals) and Pll-nanoplex/lipiodol (E, 13 animals). Seventy two hs post-injection, all the animals were anesthetized, scanned by spiral computed tomography (CT, GE Prospeed, USA) in the supine position for observation of polyplex emulsion retention in liver and then for harvesting tumors and liver samples. All the samples were then divided into four parts: ① One part were fixed in 10% neutral buffered formalin (0.1 M phosphate buffered saline) and embedded in paraffin for immunohistochemistry and histomorphometric evaluation. ② One part from each sample was fixed in methylmethacrylate and then analyzed by transmission electron microscope (TEM) and scanning electron microscope (SEM) for evaluating Cell uptake of nHAP and nanoplex. Chemical elemental mapping and energy-dispersive spectroscopy (EDS) were subsequently performed, using high-resolution SEM (Bruker Nano GmbH Berlin, Germany) equipped to EDS analyzer and operated at 20 keV in the Electronic Microscopy Laboratory of Chinese Academy of Sciences Coal Chemistry. ③ One part was analyzed by western blotting for the investigation of EGFP-wt-p53 fusion protein according to reference [1, 9, 10]. ④ One part was digested by trypsin method for parenchyma cells, whose green fluorescent fusion protein were first observed under fluorescence microscope and then analyzed by flowcytometry for transfection efficiency (TE) and mean fluorescence intensity (MFI).

### Therapeutic effects of nanoplex/lipiodol emulsion mediated combined therapy in vivo

The operations were taken by selective catheterization of the left hepatic artery and trans-arterial injection 2 ml of different emulsion per kg body weight to former described rabbits VX2 models:pDNA/lipiodol (A, 16 animals), L-nanoplex/lipiodol (B, 20 animals), U-nanoplex/lipiodol (C, 10 animals), Ca-nanoplex/lipiodol (D, 10 animals), Pll-nanoplex/lipiodol (E, 30 animals). For all the animals, blood hepatic biochemical levels of total biliflavin (TBL), aspartate aminotransferase (AST) and alanine aminotransferase (ALT) was investigated 1 day before and 1, 3, 5, 7 days after operation. The longest (L) and shortest (S) of tumor diameter was measured by spiral computed tomography (CT, GE Prospeed, USA) on dopy rabbits of each group in the supine position 1day pre-operation, 1 week and 2 weeks post-operation. The volume (V) was calculated according to the eq. V = L × S^2^/2. The tumor growth rate (TGW) was defined as (postoperative volume/preoperative volume) × 100%. All survival time of the animals were daily documented.

### Physical characterizations of nanoparticles and nanoplexs

(1): The size and polydispersity of the nanoplexs were evaluated by TEM (Osaka, Japan). (2): The zeta-potential was measured by zeta-potential analyzer (BDL-B, Shanghai) at 25 °C after diluting the dispersion to an appropriate volume with water. (3): For the DNA combination assay, 10 μl of each polyplex solutions with varying ratios of pDNA/ nanoplex mentioned above were analyzed by 1.0% agarose gel electrophoresis in Tris-Borate-EDTA buffer and visualized by SYBR Green I dye according to the protocol (Invitrogen, Carlsbad, CA, USA). (4): For the pDNA protection assay, 10 μl of each polyplex solution was first incubated with isovolumic rabbit serum at 37 °C for 12 h followed by addition of isovolumic alkaline lysis solution (0.2 N NaOH, 1% SDS). After gentle reversal and 3 min incubation at 4 °C, 7.5 μl acid solution (5 M AcO−/AcOH, pH 4.8) was added and incubated at 4 °C for 10 mins. After centrifugation at 5000 g for 10 mins at 4 °C, the supernatant was mixed with 0.6 volume of 100% dimethylcarbinol for 10 mins at − 20 °C. Following centrifugation same to the above, the pellet was resuspended in isovolumic TE buffer (10 mM Tris-Cl, 1 mM EDTA, pH 8.0). Eventually, the pDNA was purified by HiSpeed Plasmid Mini Kit (Qiagen, German) and an aliquot was analyzed by agarose gel electrophoresis.

### Statistical analysis

All data were expressed as Mean ± SD. Means between multi-groups were compared using one-way ANOVA and Fisher-LSD multiple comparison test. Survival analysis was estimated by the Kaplan-Meier survival method, with the statistical significance of survival distributions evaluated by log-rank tests. The event used as an end point was death. A *p* value of 0.05 or less was considered significant. Statistical analysis was performed using SPSS 12.0.

## Results

### Optimal dosage for safe procedure and antitumoral effect of nHAP based nanoplexs in vitro

**Fig. 1:**
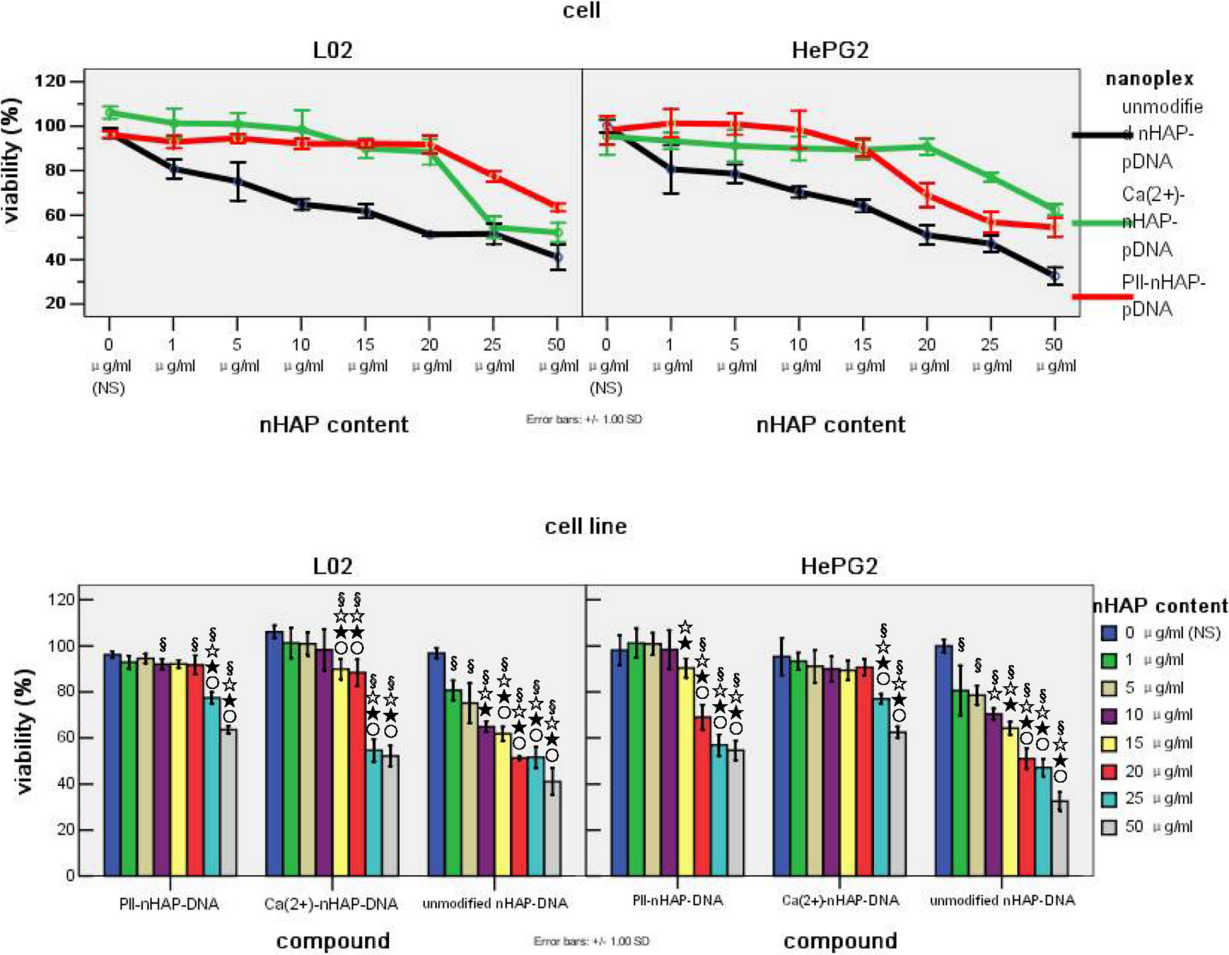
Cell viability of hepatoma HepG2 and hepatocytes L02 cell line in different concentration of three nHAP based nanoplexs. I: Comparison among different nanoplexs at same concentration. II: Comparison among various concentration of same nanoplex. Note for graphic II, §☆★○ represent significant difference from NS (control group), 1 μg/ml, 5 μg/ml and 10 μg/ml respectively as calculated with one-way analysis of variance and Fisher-LSD multiple comparison test

In general, cell viability of both cell lines decreased with increased concentration of nanoplexs. Slight L02 normal liver cell viability was decreased, whereas much more HepG2 tumor cell viability was decreased when both treated by same concentration of Pll-nHAP-PEGFP-C2 (Pll-nanoplex). The contrast were most obvious when Pll-nanoplex concentration is 15 μg/ml, striking a balance between safe transfection (about 4% reduction of L02 cell viability) and most antitumoral effect (about 30% reduction of HepG2 cell viability). For Ca-nHAP-PEGFP-C2 (Ca-nanoplex), the results were just the opposite, showing much more cytotoxicity for L02 than HepG2, especially at the concentration of 15 μg/ml. For unmodified nHAP-PEGFP-C2 (U-nanoplex), cell viability of both HepG2 and L02 were same decreased. The obvious conflicting cell viability of HepG2 versus L02 in 15 μg/ml of Ca-nanoplex and Pll-nanoplex makes us choose 15 μg/ml concentration of nanoplex for the following test (Fig. 1).

### Pll-nanoplex shows safest procedure and most effective tumoricidal activity in vitro

**Fig. 2:**
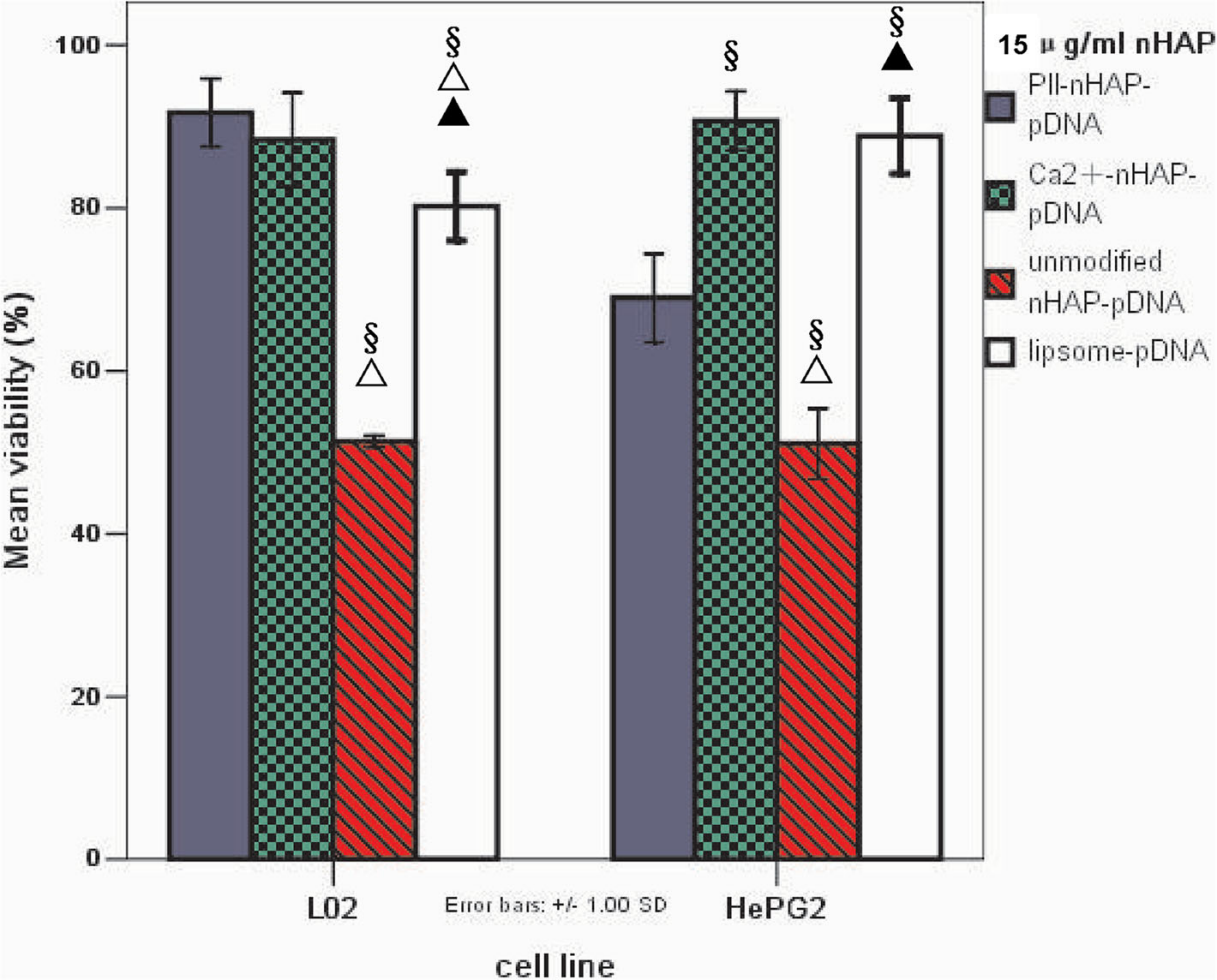
Viability comparison of among cells treated by 15 μg/ml of three nHAP based nanoplexs and L-nanoplex. Note:*△▲represent significant difference from Pll-nanoplex, Ca-nanoplex and Un-nanoplex with one-way analysis of variance and Fisher-LSD multiple comparison test

When 15 μg/ml of three nHAP based nanoplexs and liposome were compared, cell viability of HepG2 was decreased by all the four polyplexs, in the order of Ca-nanoplex> L-nanoplex >Pll-nanoplex> U-nanoplex. Cell viability of L02 was also decreased by all the four polyplexs but in the order of Pll-nanoplex, Ca-nanoplex >L-nanoplex>U-nanoplex, with no statistical significance between Ca-nanoplex and Pll-nanoplex. In all, Pll-nanoplex showed the most L02 cell viability and HepG2 tumoricidal acivity, whereas the U-nanoplex showed the least L02 cell viability and HepG2 tumoricidal effect (Fig. 2). Thus, Pll-nanoplex is the best system in vitro, taking into account safe process and antitumoral activity.

### Pll-nanoplex mediated best therapeutic effect and nHAP based gene delivery in vitro

**Fig. 3:**
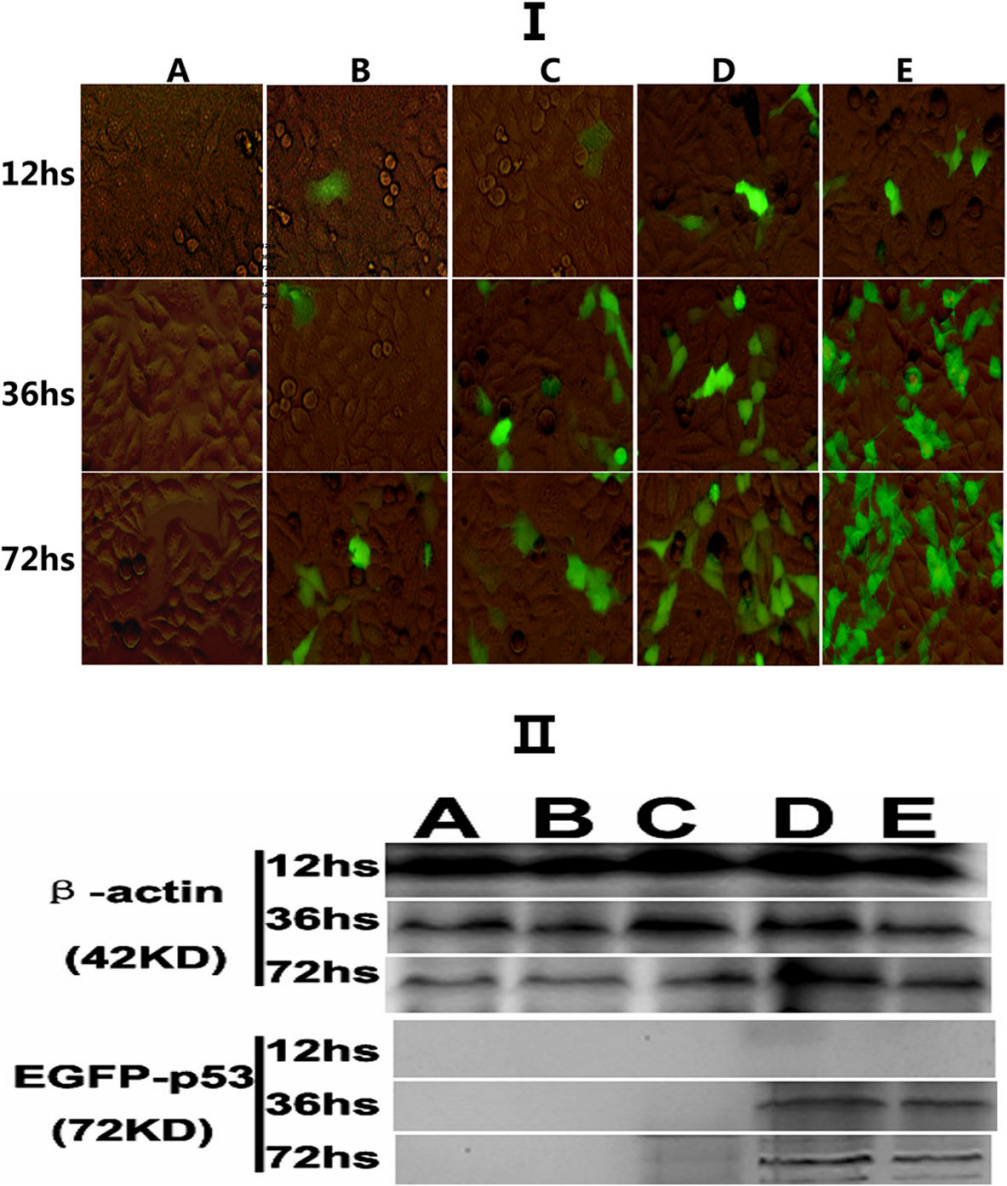
Obvious green fluorescence of transfected-positive HepG2 cells observed by fluorescence microscope (FM). **II**:Expression of EGFP-p53 protein transfected-positive HepG2 cells observed by western blot. Note: NS (normal saline+ − PEGFP-C2, A), L-nanoplex(E), Pll-nanoplex (D), Ca-nanoplex (C), U-nanoplex (B)

HepG2 cells in group NS (normal saline+ − PEGFP-C2, A) undergo unsuccessful gene transfection in the absence of transfection reagent (liposome) or nHAP carrier particles. In contrast, obvious green fluorescence of transfected-positive cells can be observed by fluorescence microscope in all the four nanoplex groups, increased with extension of time (72 hs > 36 hs > 12 hs) and was in the order of L-nanoplex(E) > Pll-nanoplex (D) > Ca-nanoplex (C) > U-nanoplex (B) at same Observation time points (Fig. 3I). Transfection efficiency (TE) and mean fluorescence intensity (MFI) was then analyzed by flowcytometry for HepG2 cells. Both TE and MFI of all groups increased in parallel with time (72hs > 36hs > 12hs) and increased in the order of E > D > C > B > A for different polyplexs at same Observation time points. However, group D and E showed statistically significant higher TE, MFI, apoptosis and necrosis rates than other groups. The liposome showed the highest TE and MFI, whereas Pll-nanoplex induced the most apoptosis and necrosis of HepG2 cell at 36 and 72 hs, respectively, significantly compared to the other three nanoplexs (P<0.05). As for apoptosis and necrosis analysis, PEGFP-C2-wt-p53 is used instead of PEGFP-C2 (Table 1). For the target gene expression, the expression of EGFP-wt-p53 fusion protein only be detected by in L-nanoplex(E) and Pll-nanoplex (D) group at 72 hs and 36 hs (Fig. 3 **II**).

**Table 1:** Transfection efficiency (TE), mean fluorescence intensity (MFI), apoptosis rate (AR) and necrosis rates (NR) of HepG2 cells analyzed by flowcytometry in vitro: pDNA (A),U-nanoplex (B), Ca-nanoplex (C), Pll--nanoplex (D), L-nanoplex (E). All the data were calculated with one-way analysis of variance and Fisher-LSD multiple comparison tests

		Group A	Group B	Group C	Group D	Group E
TE (%)	12hs	0	0.1 ± 0.05	0.1 ± 0.10	0.1 ± 0.08	0.7 ± 0.10^a^
	36hs	0	0.1 ± 0.06	0.3 ± 0.08	2.1 ± 0.26^a,b,c^	20.1 ± 1.53^a,b,c,d^
	72hs	0	0.2 ± 0.02	0.4 ± 0.14	6.3 ± 0.33^a,b,c^	16.8 ± 1.48^a,b,c,d^
MFI	12hs	86.4 ± 7.22	88.0 ± 5.61	89.5 ± 2.70	96.5 ± 16.00	93.8 ± 3.56
	36hs	90.3 ± 2.80	86.3 ± 5.59	93.3 ± 3.77	106.7 ± 10.49^a,b,c^	189.9 ± 10.03^a,b,c,d^
	72hs	85.4 ± 2.68	97.2 ± 4.62	95.3 ± 3.53	135.4 ± 17.10^a,b,c^	143.2 ± 17.66^a,b,c,d^
AR (%)	36hs	0.2 ± 0.08	5.0 ± 1.47^a^	0.4 ± 0.06^b^	6.5 ± 0.71^a,b,c^	2.0 ± 0.57^a,b,c,d^
	72hs	1.7 ± 0.58	2.5 ± 0.75	1.85 ± 0.28	36.0 ± 1.70^a,b,c^	24.6 ± 1.93^a,b,c,d^
NR (%)	36hs	0.8 ± 0.17	1.7 ± 0.48	1.0 ± 0.06	6.8 ± 0.64^a,b,c^	9.8 ± 3.38^a,b,c,d^
	72hs	2.1 ± 0.41	3.2 ± 0.89	2.6 ± 0.41	15.3 ± 4.08^a,b,c^	18.0 ± 10.92^a,b,c,d^

^a,b,c,d^represent significant difference from group A, B, C, D respectively

### Only Pll-nanoplex/lipiodol emulsion selectively targeted and successfully transfer gene to VX2 tumor

**Fig. 4:**
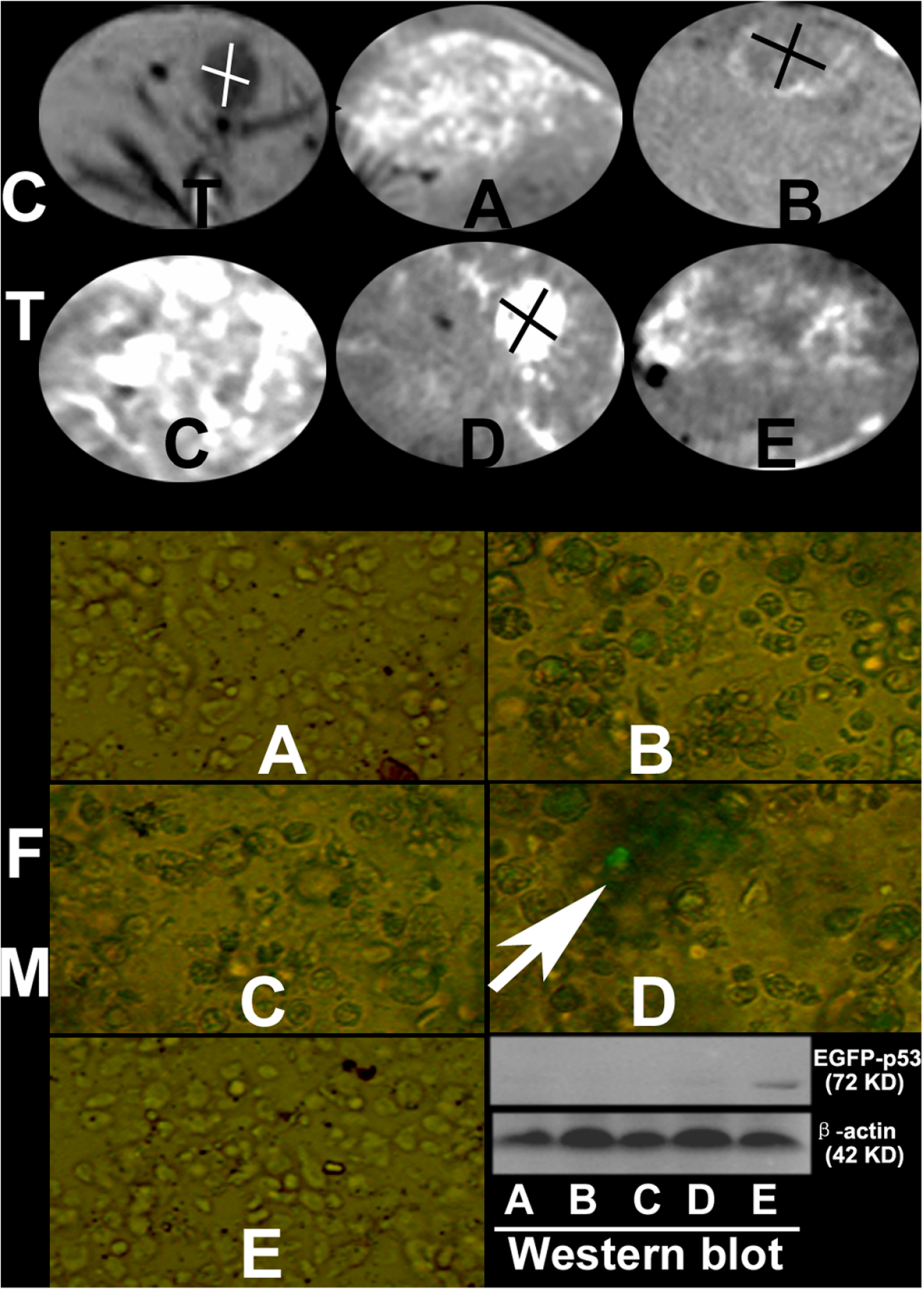
VX_2_ tumor can be shown clearly by CT on the left lobe of liver (T, area showed by white cross) before emulsion injection. After in vivo intra-arterial injection of PEGFP-C2-wt-P53/lipiodol (A), L-nanoplex/lipiodol (E), U-nanoplex/lipiodol (B), Ca-nanoplexCa-nanoplex/lipiodol (C), Pll-nanoplex/lipiodol (D), nanoplex emulsion in group D displayed significantly stronger and more selectively deposits in tumor area (D, area showed by black cross), compared to the slight but selective deposits in group B (B, area showed by black cross), whereas emulsions in group A, C, E produced no tumor-selective retention potency but diffuse distribution in liver. In contrast to group A, B, C and E, EGFP-wt-P53 expression was observed by fluorescence microscope (FM) for green fluorescence (the arrow) and by western blot for a ∼ 72 kDa molecular weight band only in tumor of group D

**Fig. 5:**
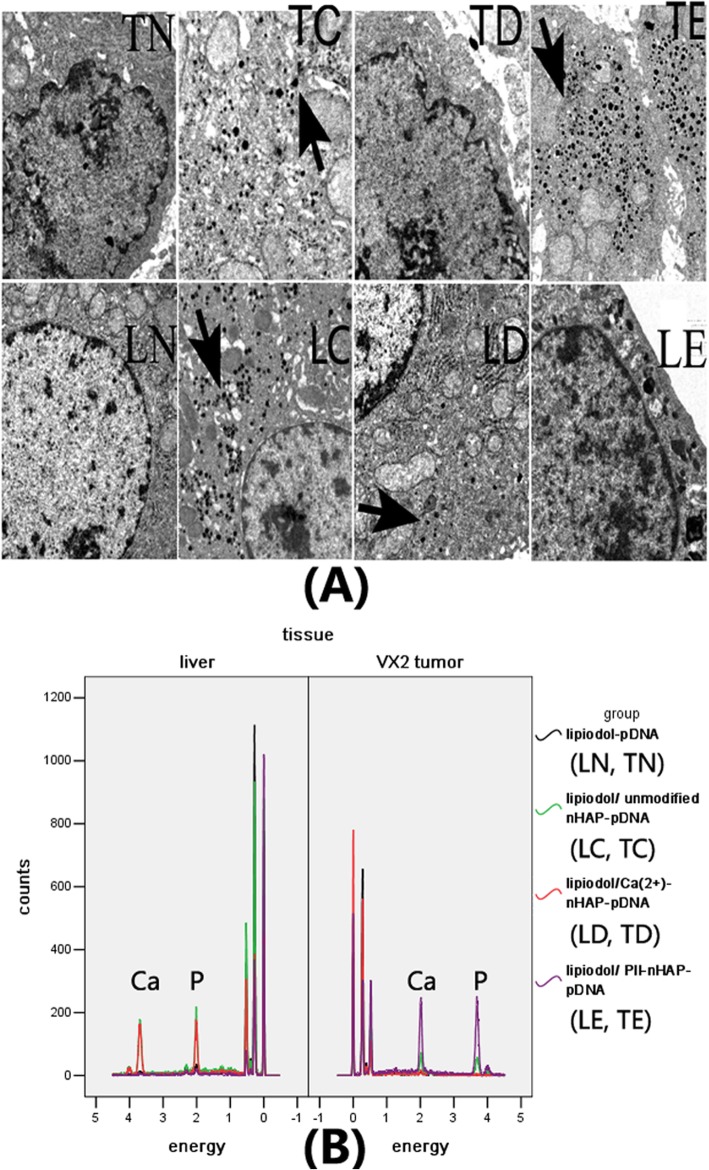
**a** Observation of nHAP presence (small black spots showed by arrows) under transmission electron microscopy (TEM) with magnification of 25,000 times after in vivo intra-arterial injection of polyplex/lipiodol emulsion to VX_2_ tumor-bearing Rabbits: no nHAP deposit in VX_2_ tumor cell (TN) and normal liver cell (LN) of PEGFP-C2-wt-P53/lipiodol group. nHAP deposit in both VX_2_ tumor cell (TC) and normal liver cell (LC) of U-nanoplex/lipiodol group. nHAP deposit in cytoplasm of normal liver cell (LD) but VX_2_ tumor cell (TD) of Ca-nanoplexCa-nanoplex/lipiodol group. nHAP can selectively deposit in cytoplasm of VX_2_ tumor cell (TE) but normal liver cell (LE) of Pll-nanoplex/lipiodol group. **b** Semi-qualitative energy dispersive spectroscopy (EDS) spectra of all the tissues above in Fig. 5a were investigated under scanning electron microscopy (SEM): As presented, their spectra have been overlapped except in the region of 2.010 and 3.692 keV which represent the calcium and phosphorus element respectively. The peak area of calcium and phosphorus element can be seen in the samples of TC, LC, LD, TE but LN, TN, TD, LE. The main components of nHAp were calcium and phosphorus in the molar ratio Ca/P around of 2.0, which is similar to the estimated Ca/P molar ratio of TC, LC, LD, TE . In contrast, the Ca/P molar ratios of LN, TN, TD, LE had similar consequences around 0.6. The EDS analysis further confirm presence of nHAPs shown in Fig. 5a. Therefore, the existence of nHAP was confirmed in the samples of TC, LC, LD, TE but LN, TN, TD, LE

**Fig. 6:**
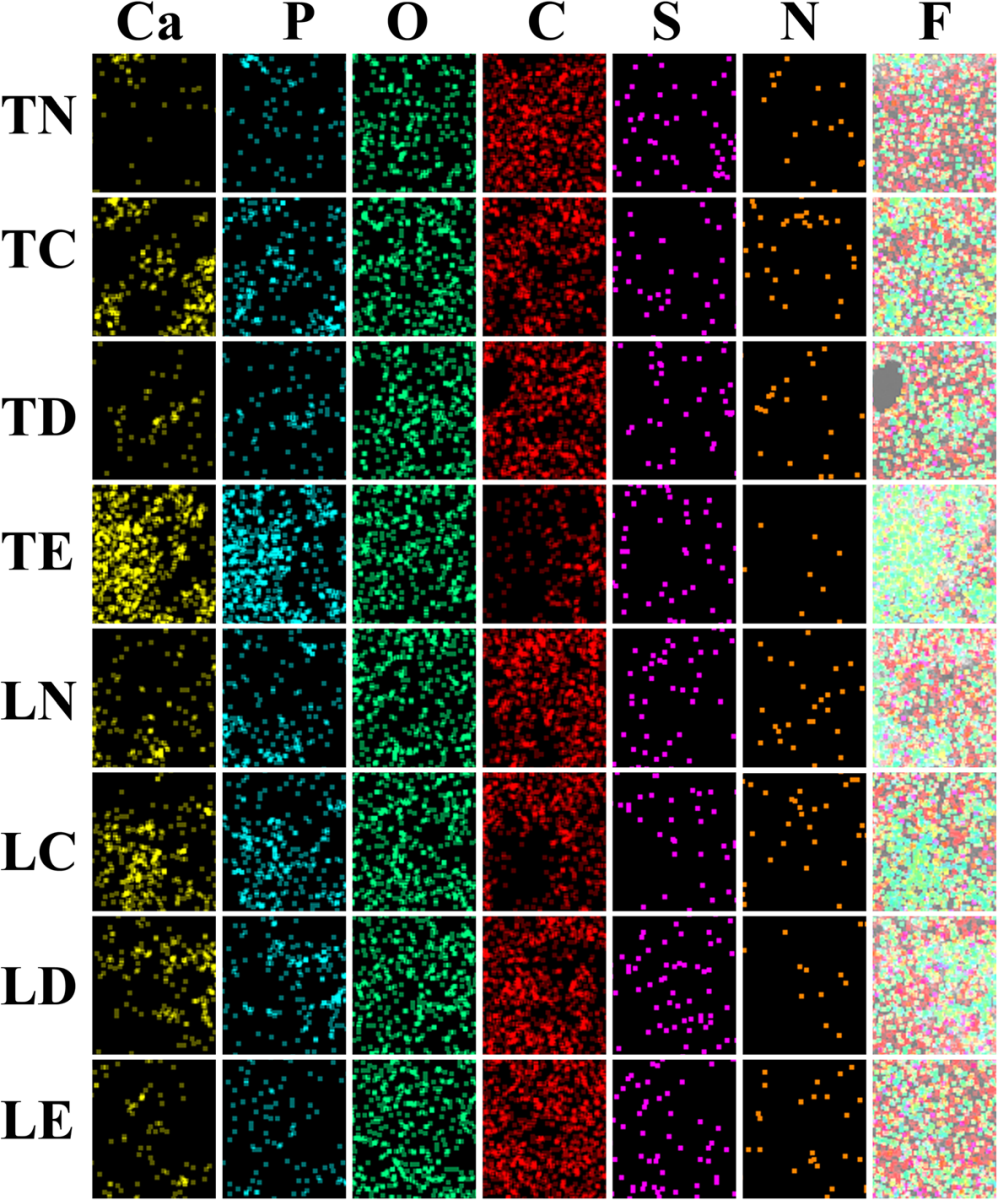
Furthermore, elemental mapping examination has shown the abundant presence of element Calcuim (Ca) and phosphorus (P) in TC, LC, LD, TE (with the order of TE > LC > TC > LD), while these observations were not observed in LN, TN, TD, LE. Element Oxygenium (O), Carbon (C), Sulfur (S), Nitrogen (N) present in all tissues show no obvious difference. F represent the fusion image of all element above in tissue

The successful transfer of wt-p53 into HepG2 cell line in vitro could not recapitulate all the necessary process that happen in HCC in vivo. We therefore sought to address this concern by applying nanoplexs/lipiodol in rabbit VX2 hepatic cancer model. For target gene expression, western blot showed that the expression of EGFP-wt-p53 fusion protein only be detected by in tumor cells of Pll-nanoplex/lipiodol group, whose obvious green fluorescent of also be observed from fluorescent microscope (Fig. 4). Subsequent flowcytometry showed that TE and MFI of tumor cells in Pll-nanoplex/lipiodol group were significantly higher than other groups (Table 2). Transverse CT scan (Fig. 4) revealed that the specific retention of nanoplex/lipiodol emulsions in implanted VX2 tumor 72 hs after the transarterial delivery, increased with decreased diffuse in liver and was in the order Pll-nanoplexPll-nanoplex/lipiodol (D)U-nanoplex/lipiodol (B) > lipiodol (A), liposome-wt-p53/lipiodol (E), Ca-nanoplex/lipiodol (C). In fact, group A, E, C showed no selective retention in tumor (Fig. 4). For the nanoparticle distribution, TEM, EDS and subsequent elemental mapping all showed that the Pll-nHAP can only be observed in the cytoplasm of tumor cells but liver cells, whereas the Ca-nHAP can only be observed in the cytoplasm of the liver cells but tumor cells, and the unmodified nHAP can be observed in both tumor and liver cells (Fig. 5, Fig. 6).

**Table 2:** Flowcytometry was utilized to measure and normalize transfection efficiency (TE) and mean fluorescence intensity (MFI) of harvested tumor cells across different groups in vivo: pDNA/lipiodol (A), L-nanoplex/lipiodol (E), U-nanoplex/lipiodol (B), Ca-nanoplex/lipiodol (C), Pll-nanoplex/lipiodol (D)

	Group A	Group E	Group B	Group C	Group D
TE (%)	0.1 ± 0.06	0.2 ± 0.06	0.2 ± 0.07	0.2 ± 0.07	4.1 ± 0.64^a,b,c,d^
MFI	95.6 ± 4.71	106.5 ± 11.15	05.3 ± 9.27	100.2 ± 12.39	124.4 ± 17.23^a,c,d^

^a,b,c,d^ represent significant difference from group A, E, B, C respectively (*P* < 0.05) . The almost 0% transfected cells in group A exhibit strong autofluorescence, which attributes to the high background fluorescence. However, group E have more MFI due to the enormous green fluorescent of EGFP-wt-P53 fusion protein in its 4% pEGFPC2-wt-P53 positive transfected cells. All the data were expressed as mean ± SD and calculated with one-way analysis of variance and Fisher-LSD multiple comparison tests

### Pll-nanoplex/lipiodol emulsion mediated the most effective procedure safely in vivo

#### Overall tumor volumes

As shown in Table 3: There were no significant difference among all groups in preoperative overall tumor volume (P^A VS E^ = 0.282, P^A VS B^ = .054, P^A VS C^ = .344, P^A *VS*D^ = .081, P^E VS B^ = .274, P^E VS C^ = .958, P^E VS B^ = 0.526, P^B VS C^ = 0.367, P^B VS D^ = 0.508, P^C *VSB*^ = 0.656). One week after trans-arterial administration of different nanoplex/lipiodol emulsions, significant smaller tumor volume were observed in group E than other groups (P^A VS E^ = 0.598, P^A VS B^ = .057, P^A VS C^ = .834, P^A VS B^ = .000, P^E VS B^ = .125, P^E *VSC*^ = .812, P^E VS B^ < 0.001, P^B VS C^ = 0.125, P^B VS D^ = 0.009, P^C *VSB*^ < 0.001). Two weeks after operation, trans-arterial administration of B and D led to significant delay of tumor growth than group A, E, C (P^A VS E^ = 0.797, P^A VS B^ = .000, P^A VS C^ = .894, P^A VS D^ = .000, P^E VS B^ = .000, P^E VS C^ = .934, P^E VS D^ < 0.001, P^B VS C^ < 0.001, P^C VS D^ < 0.001). In addition, no smaller tumor volume was noted in Group E than group C 2 weeks after operation(P^B VS D^865).

**Table 3:** Preoperational and postoperational VX2 tumor volume (mm^3^, mean ± SD) of different groups: pDNA/lipiodol (A), L-nanoplex/lipiodol (E), U-nanoplex/lipiodol (B), Ca-nanoplex/lipiodol (C), Pll-nanoplex (D). All the data were calculated with one-way analysis of variance and Fisher-LSD multiple comparison tests

Groups	preopertion	1 w postopertion	2 w postopertion
A	1257.8 ± 259.49	1937.7 ± 691.15	3873.2 ± 1632.08
E	1169.9 ± 264.69	1860.2 ± 520.80	3789.2 ± 991.56
B	1066.6 ± 220.95	1598.3 ± 323.04	2010.6 ± 546.49^a,b^
C	1164.9 ± 258.87	1900.6 ± 375.93	3820.6 ± 1059.55^c^
D	1125.4 ± 216.84	1168.6 ± 177.51^a,b,c,d^	1950.1 ± 417.13^a,b,d^

^a,b,c,d^represent significant difference from group A, E, B, C respectively (*P* < 0.05)

#### Tumor growth rate (TGW)

**Fig. 7:**
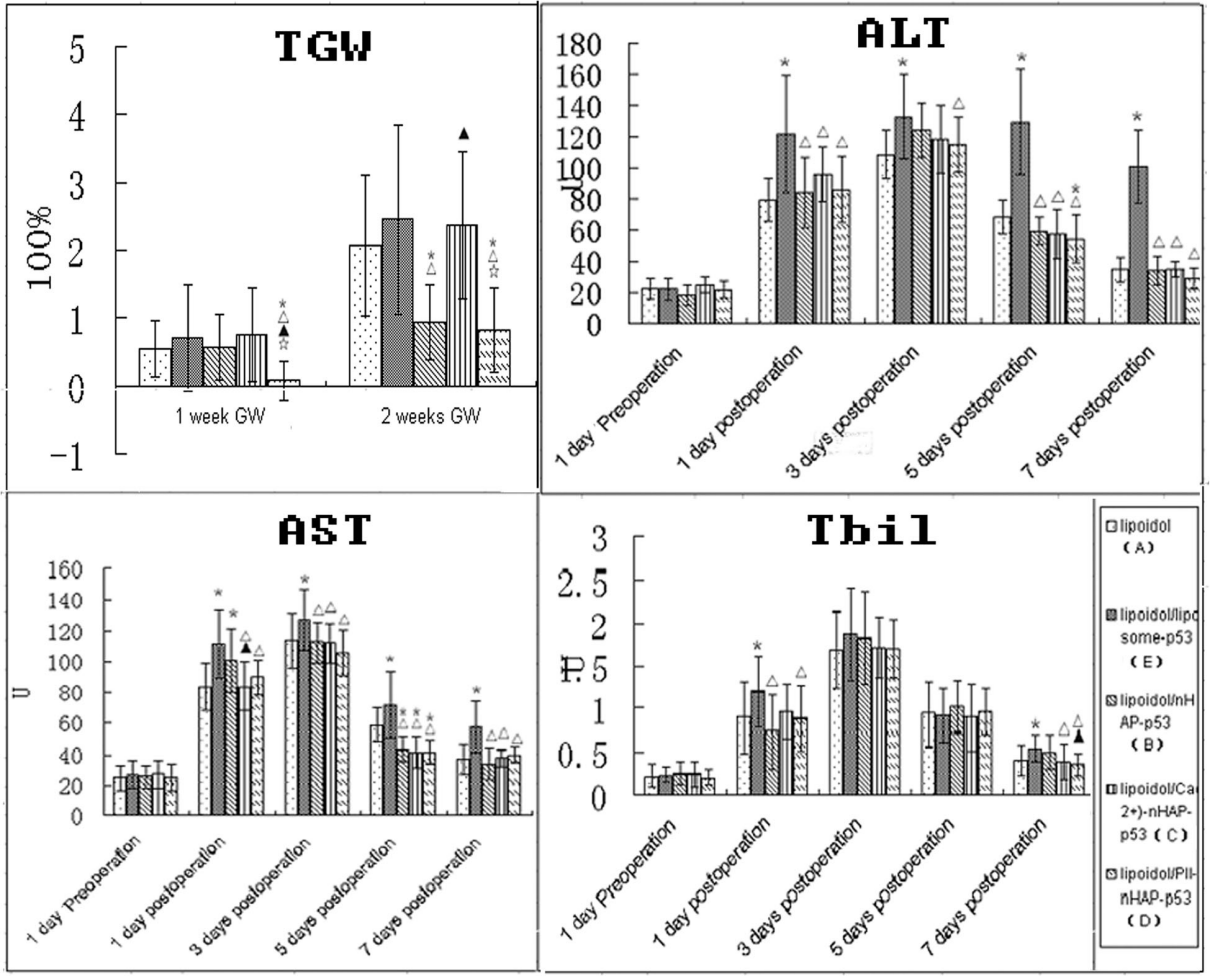
Tumor growth rate (TGW), plasma levels of total biliflavin (TBL), aspartate aminotransferase (AST) and alanine aminotransferase (ALT) in different groups: PEGFP-C2-wt-P53/lipiodol (A), L-nanoplex/lipiodol (E), U-nanoplex/lipiodol (B), Ca-nanoplex/lipiodol (C), Pll-nanoplex/lipiodol (D). *△▲☆ represent significant difference from group A,E,B,C respectively as calculated with one-way analysis of variance and Fisher-LSD multiple comparison test

For all groups, TGW of all groups increased with the extension of time (2 weeks> 1 week). However, 1 week TGW of only group D is statistically significant more than other groups. Two weeks TGW of group B and D were statistically significant more than other groups. Group D has the least 2 weeks TGW. The overall tumor growth changes revealed that Pll-nanoplex/lipiodol emulsion can inhibit the one and two-week significantly more than the others (Fig. 7). Group E and C inhibited the least tumor growth than the remaining 3 groups in vivo.

#### Hepatic function investigation

There is no significant difference in all groups for the plasma levels of TBL, AST and ALT before operation. One day postoperation: group E exhibit enhanced ALT and TBL compared to other groups. Group E and B exhibit enhanced AST compared to other groups. Three days postoperation: group E exhibit enhanced ALT and AST compared to other groups. Group E and B exhibit enhanced AST compared to other groups. There is no significant difference in all groups for the plasma levels of TBL. Five days postoperation: group E exhibit enhanced ALT than other groups and group D exhibit lower ALT than group A. Group A exhibit less AST compared to all other nanoplex groups. There is no significant difference in all groups for the plasma levels of TBL. Seven days postoperation: group B exhibit enhanced TBL, AST and ALT than all other groups. In all, contrast to the severe hepatic function damage of liposome/lipiodol, all the nHAP based emulsion enhanced the plasma levels of liver markers transiently but all recovered within 1 week post operation, except the slightly increased Tbil of Ca-nanoplex/lipiodol group (Fig. 6). So nHAP/lipiodol based emulsion is same safe for long term hepatic function (Fig. 7).

#### Survival benefit

**Fig. 8:**
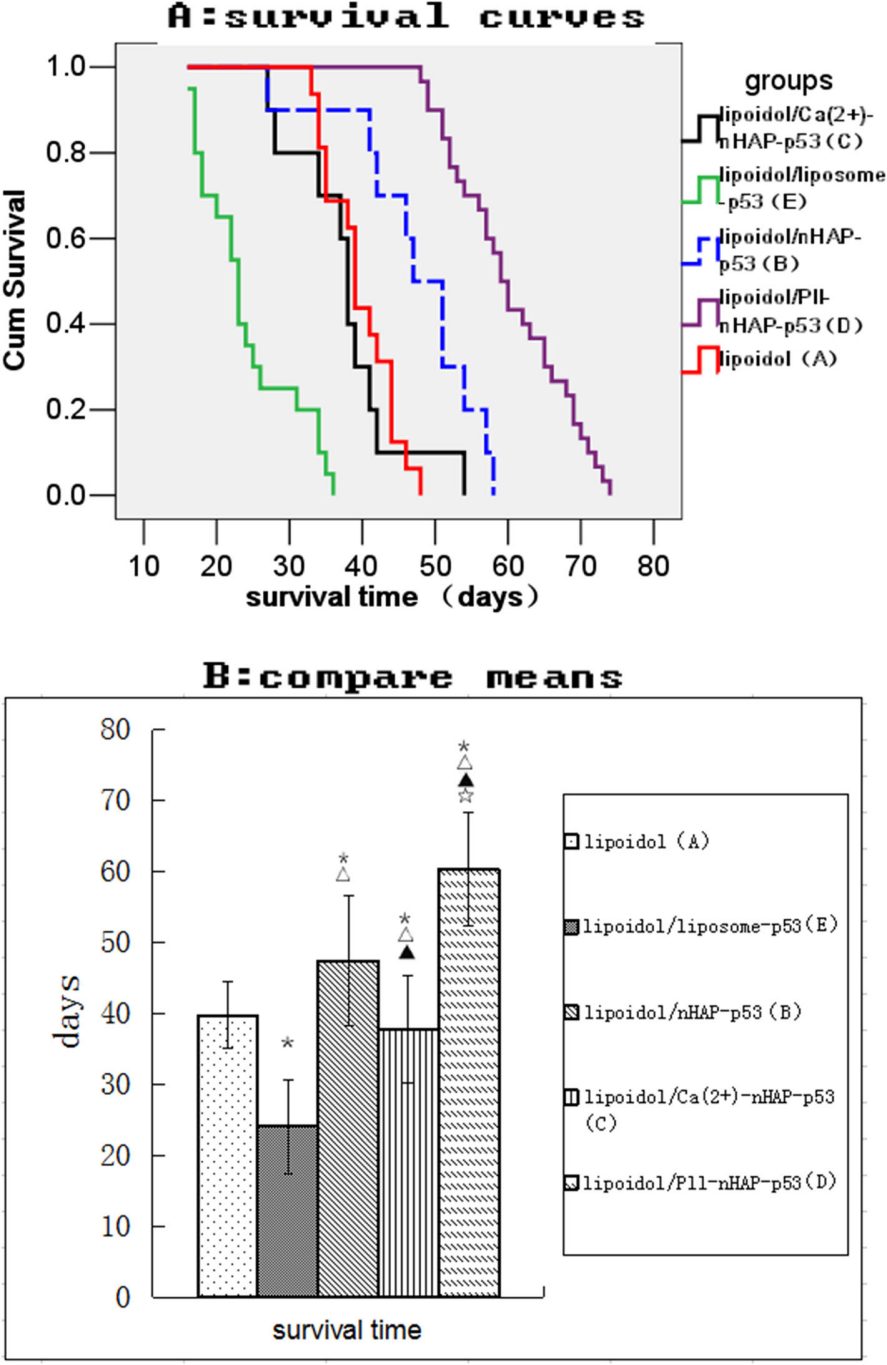
Overall survival curves (**a**) and survival time (**b**) of animals from different groups. *△▲☆ represent significant difference from group A,E,B,C respectively as calculated with one-way analysis of variance and Fisher-LSD multiple comparison test

Log-rank test for Kaplan-Meier curves denied the null hypothesis “all survival curves are the same”. Further pairwise comparison show that, compared to group A, significant longer survival time can be observed in group B (*p* = 0.002) and D (*p* < 0.001) while significant shorter survival time can be observed in group E (p < 0.001). There is no significant difference for the survival time between the Group A and C (*p* = 0.591). Group D can significantly enhance the survival benefit than Group B (p < 0.001). Group D enhance the most survival benefit (Fig. 8a). The survival time (mean ± SD) for group A, E, B, C, D are 39.7 ± 4.69, 24.1 ± 6.61, 47.4 ± 9.20, 37.8 ± 7.60 and 60.4 ± 7.99 days, respectively (Fig. 8b).

In all, Pll-nanoplex/lipiodol supplied to the best therapeutic effect without severe influence of hepatic function, whereas liposome/ lipiodol emulsion resulted in the least survival benefit with most severe influence of hepatic function despite of its good inhibition of tumor growth in 2 weeks (Fig. 7).

### Surface modified nHAP with pll became cationic and much smaller

**Fig. 9:**
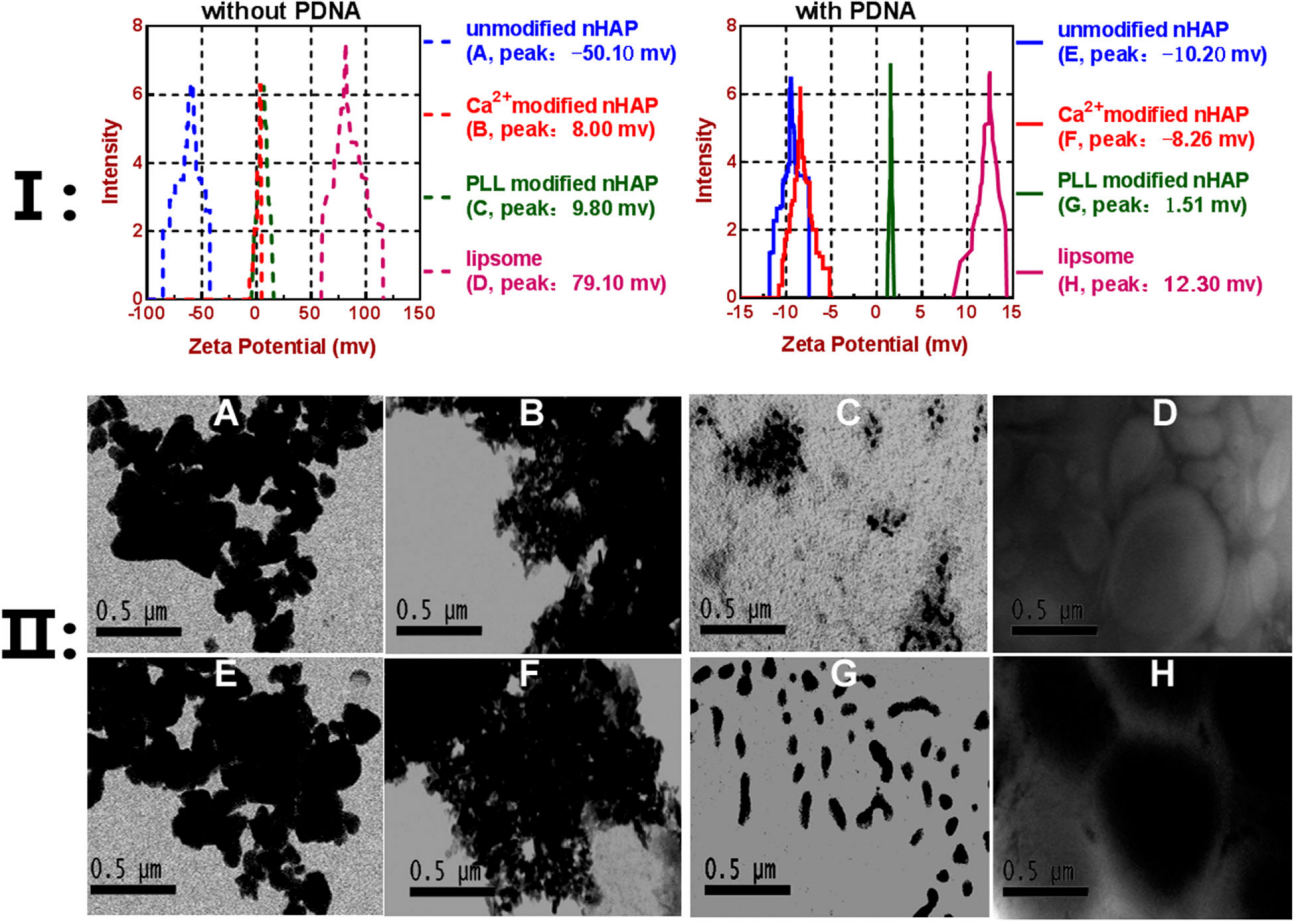
Zeta-potential and sizecomparation of various nanoplexs under zeta-potential analyzer (**I**) and transmission electron microscopy (TEM) with magnification × 25,000 (**II**) respectively

**I:** As for the zeta-potential, both lipsome and Pll modification can turn very negatively charged nHAP to slightly cationic nanoplex (Fig. 9I). In all, Only Pll-nHAP can form cationic nanometeric nanoplex with pDNA. **II**: Unmodified nHAP (A) and unmodified nHAP-PEGFP-C2-wt-p53 complex (E) can easily congregated into large particles of 251 ± 53.6 nm and 282 ± 65.9 nm in diameter respectively. Ca^(2+)^ modified nHAP (B) and Ca-nHAP-PEGFP-C2-wt-p53 complex (F) crystallized to much larger particles of 851 ± 651.2 nm and 883 ± 658.7 nm in diameter respectively, even precipitate with very slight water solubility. Pll modified nHAP (C) disperse with small particles of 15 ± 3.2 nm but easily congregated, whereas Pll-nHAP-PEGFP-C2-wt-p53 complex (G) scattered and keep even small particles of 97 ± 13.2 nm in steady solution. Lipsome (D) and lipsome-PEGFP-C2-wt-p53 (H) complex scattered and keep big particles of 555 ± 63.2 nm and 658 ± 71.8 nm respectively. So, TEM results showed only the Pll-nHAP–pDNA nanoplex can keep the diameter below 100 nm when any of the others either can’t form real nanoplex or the one smaller than 500 nm (Fig. 9 II).

### Only Pll-nHAP can combine and protect the most pDNA

**Fig. 10:**
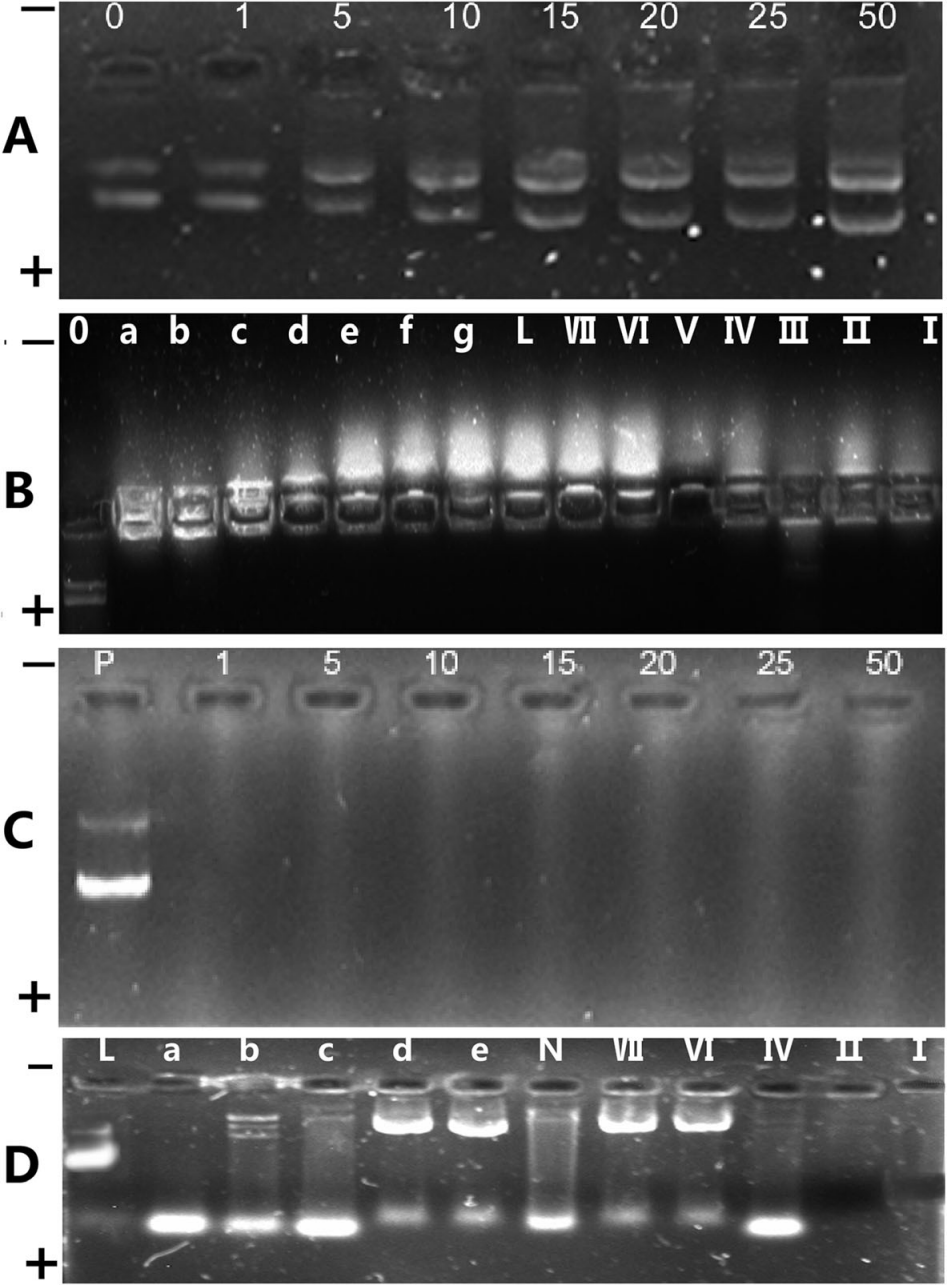
PDNA combination (A, B) and protection (C, D) effects of different nanoplex: 0, 1, 5, 10, 15, 20, 25, 50 represent unmodified nHAP /PDNA mass ratio. a, b, c, d, e, f, g represent Pll-nHAP /PDNA mass ratios of 1, 5, 10, 15, 20, 25, 50 respectively. I, II, III, IV, V, VI, VII represent Ca ^(2+)^-nHAP /PDNA mass ratios of 1, 5, 10, 15, 20, 25, 50 respectively. L and N represent liposome/PDNA complex and nude PDNA respectively. P represent PDNA without enzymes

Gel retardation experiment (Fig. 10) show that, contrary to U-nanoplex’s disability of pDNA absorption and protection, the positive charged Pll-nanoplex (Pll-nHAP /pDNA mass ratio more than 15), Ca-nanoplex (Ca-nHAP/pDNA mass ratios more than 25), liposome/pDNA complex exhibited strong potency of pDNA absorption and protection from the destruction of nucleinase in rabbit serum. Pll-nanoplex can absorb and protect more pDNA than Ca-nanoplex when same nHAP was used, which may explain its stronger capability of pDNA transfection efficiency.

### No significant differnece for water-in-oil percentage [W/O], droplet sizes and viscosity of different emulsion

As shown in Table 4, there is no significant difference for the mean percentage of water-in-oil [W/O], droplet sizes and viscosity for different emulsion: pDNA/lipiodol (A), L-nanoplex/lipiodol (E), U-nanoplex/lipiodol (B), Ca-nanoplex/lipiodol (C), Pll-nanoplex (D).

**Table 4:** Mean percentage of water-in-oil [W/O], droplet sizes and viscosity for different emulsion: pDNA/lipiodol (A), L-nanoplex/lipiodol (E), U-nanoplex/lipiodol (B), Ca-nanoplex/lipiodol (C), Pll-nanoplex (D)

Groups	W/O (%)	Droplet Size (μm)	Viscosity (cP)
A	65.5 ± 3.23	30.5 ± 3.08	141.6 ± 1.36
E	67.9 ± 4.69	30.2 ± 2.89	138.2 ± 1.58
B	66.6 ± 2.91	28.3 ± 3.08	140.6 ± 2.43
C	64.8 ± 2.82	30.6 ± 3.09	139.6 ± 3.05
D	65.4 ± 2.32	29.6 ± 3.01	139.1 ± 2.91

## Discussion

Our former reports [1, 9, 10, 14] successfully innovated TAE-gene therapy for hepatocellular carcinoma (HCC) through application of Pll-nanoplex. This study focus on comparing and investigating the crucial physico-chemical characterizations of four nanoplexs that give better therapeutic effect and more safety for nano-TAE gene therapy. The purpose of this new therapy is to combine the antitumoral effect of nanoparticle, target gene therapy and transarterial embolization (TAE) through application of one system. So, all that three requirements must be satisfied when searching the proper systems for HCC treatment.

First, the nanoplex must have specific anti-tumor activity. Among various non-viral gene carriers, liposome remain most efficient and prevalent to date. However, general serious toxicity to the cell membrane [15, 16] makes it hard to have specific antitumoral effect. HAP, with molecular formula Ca10(PO4)6(OH)2, is the essential component of human enamel [17–19] and its nanoparticle (nHAP, 0.1-100 nm in diameter) proved to have good tissue compatibility both in vitro and vivo [20–23]. However, that safety is only observed in bone tissue and nonparenchymal cell. In the present study, the unmodified nHAP showed comparable cytotoxicity both to HepG2 and L02 cells, mostly due to its surface properties as well as high negative zeta-potential, whose inner expulsion also induce nHAP precipitation and congregation [18, 21, 24]. As surface coating is a primary determinant of cytotoxicity, nHAP was surface-modified by utilizing Ca^(2+)^ and Pll, representing popular strategies of inorganic and organic respectively. For Ca-nHAP nanoplex, the particles precipitate to microparticles right after the Ca^(2+)^ addition and the big particles definitely cover up the cell membrane and may influence the normal substance exchange, the main reason for its nonspecific cytotoxicity. As expected, Pll-nanoplex obviously inhibited the proliferation of hepatoma cells whereas proliferation of normal hepatocyte was relatively slightly affected, which coincide with the report about TIO2 (titanium oxide) nanoparticles [25]. Contrary to liposome, U-nHAP and Ca-nHAP nanoplex, we attribute the privileges of Pll-nanoplex to its nanomentric diameter and slightly positive organic surface, which have stronger affinity for cell membranes to accomplish the endocytosis process. As organic molecule with strong affinity for cell membrane, Pll incorporation reduce nHAP diameter and cationize its surface, which in turn favor the interaction of nHAP to cell membrane and the following phagocytosis by tumor under physical conditions. In addition, the different phagocytosis capability of cancer and normal cell may also account for that phenomenon. After phagocytosis, the nanoparticle can distribute in cytosolic organelles and elevate its Ca^(2+)^ concentration and in turn induce tumor apoptosis by Ca^(2+)^-dependent endonuclease activation [26–28]. Take together, specific antitumoral effect may be better achieved by particles with organic surface, proper size (about 100 nm) and positive superficial zeta-potential (about +10mv) to favor the swallow of tumor cell but normal cell. In this way, we can turn cytotoxicity of nanoparticles to specific antitumoral effect [29, 30].

Second, effective gene transfer need an ideal vector to deliver naked pDNA into cells. pDNA condensation is the first step for the vector mediated gene delivery [31]. The features of large surface and high surface energy of nHAP hold strong DNA binding potency. The unmodified nHAP, however, with very negative zeta-potential value, may repel pDNA of same negative potential and thus inhibited the formation of nHAP-pDNA nanoplex, accounting for the subsequent gene delivery failure. So, the nanoplex need cationic surface to bind pDNA of negative potential by the law of opposite charges attract. For that reason, liposome, Pll-nHAP and Ca-nHAP successfully compacted the pDNA and formed nanoplexs in this study. After that, synthetic material employed for gene delivery should be or become cationic for a higher affinity for the negatively charged cytoplasm membrane followed by endocytosis [32–34]. Obviously, all the three above satisfy this requirement. Ca^(2+)^ have been demonstrated to be the most potential surface improver for nHAP [35]. However, the cationic improvement for nHAP was too poor to keep positive potential of Ca-nanoplex at same concentration (Ca-nHAP/pDNA mass ratio less than 20). In addition, Ca^(2+)^ modification promoted congregation and fusion of nHAP, which in turn decreases their surface area, porosity and results in particle bigger, less stable in emulsion and reduced absorption to pDNA. Moreover, microparticles of Ca-nHAP is too big to be swallowed by the cells, let alone the following gene transfer. The reason may be that bivalent cations, such as Mg^(2+)^, Ca^(2+)^ and Zn^(2+)^ atoms [36–38] may bond to PO4^(3−)^ ionic group of nHAP as tri-calcium phosphate (TCP, Ca_3_(PO_4_)_2_), which in turn changes the microstructure of nHAP, reduces its crystallinity of structure, increase its particle size, as well as promoting its congregation and precipitation. So, Ca^(2+)^ modification is not suitable for nHAP gene therapy. The liposome can be swallowed by the cells in vitro but its diameter (about 500 nm) is also too big to penetrate the barrier between blood and tumor cells during the processes before when endocytosis can possibly occurs in vivo [24, 39, 40]. Similar transfection failure of particles bigger than 250 nm were obtained by synergism of PEI and liposome [41] and this diameter is proved to selectively target Kupffer cells but the tumor parenchyma cell [42], indicating that similar system can’t mediated effective gene therapy to HCCs. In the present study, Pll of organic polymer, known for pDNA loading and protection, was also used for the nHAP modification. As expected, the cationic nHAP-Pll-nanoplex successfully absorbed and condensed the pDNA into polyplex below 100 nm. Similar to reports of other cell lines in vitro [43], nHAP mediated transfection efficiency to HepG2 was much lower than that of commercial liposome products such as lipofectamine 2000 in this study. However, only Pll-nanoplex can successfully transfer pDNA to rabbit VX2 tumor in vivo due to its small enough diameter(< 100 nm) and cationic, organic polyer surface, which is easier for cell to adhere. Similar to that presented here, Zauner [44] observed internalization of only few particles of polystyrene microsphere of > 100 nm in Hepa and HepG2 cell line. So, to exploit the potential of the complex mediated gene delivery for HCC in vivo, we suggest pDNA entrapment into a cationic nanometric nanoplex with organic surface (about 100 nm) as the prerequisite criteria.

Third, the specific deposition and retention of the complexes in HCC is also necessary due to the reason that all the antitumoral factors, including gene therapy, TAE and nanoparticle, need long enough time to be fully exploited in the local tumor site. Lipiodol can selectively stagnate in HCCs as different time required for its removal from normal capillaries and tumor neovasculature [45]. Trans-arterial injection of nHAP/lipiodol emulsion successfully achieve tumor embolism, target retention of nHAP in tumor and subsequent inhibition of tumor growth. In this study, the specific deposition of lipiodol and nHAP only in the tumor site was observed in Pll-nanoplex by CT images and TEM. Subsequent energy-dispersive spectroscopy (EDS) confirmed specific existence of Pll-nHAPs in VX2 tumor site. The liposome couldn’t absorb the lipiodol and develop a integral liposome-based composite, maybe due to its nonporous fat-soluble surface. That diffused localization of lipiodol and liposome do no help to the specific stagnation of liposome-pDNA in tumor. The Ca-nanoplex can indeed absorb the lipiodol and integrated into one component, but the micrometer particle can easily block the big vessel and make the lipiodol contraflow to nearly the whole liver, as illustrated in Fig. 9. The unmodified nHAP/lipiodol was observed in the tumor target of CT images. However, TEM and EDS result show that more nHAP distribute in the liver cells than in the tumor cells, suggesting that the lipiodol in fact may be eliminated by liver but stagnate in the tumor. Energy-dispersive X-ray spectroscopy (EDS) is an analytical technique used for determining the presence of chemical elements in a sample and their relative abundance. Its characterization capabilities are due to the unique atomic structure of each element that can generate a unique set of peaks on its electromagnetic emission spectrum after excited by the incoming beam of X-ray. Electron beam excitation and detection is processed under scanning electron microscopes (SEM) and transmission electron microscopes (TEM). However, of particular note, elemental mapping and EDS wasn’t be performed with TEM in this study due to its high working temperature environment (about 200 °C) operated at 200 keV, which obviously may burn the tumor tissue. With regard to the subcellular distribution, nHAP can distribute in the cytoplasm in the present study, similar to Radoslav’s result of rat pheochromocytoma PC12 cells [28].

Fourth, it’s well admitted that the advantages of water-in-oil [W/O] emulsion to oil-in-water [O/W]) emulsion in embolic effect and longer tumor retention of conventional trans-arterial chemoembolization for hepatocellular carcinoma, due to its higher viscosity, drug carriage capacity; and a longer drug release time [46]. All the four emulsions here has similar W/O percentage, droplet sizes and viscosity. So, there was no significant difference in the impact of each gene vector system on that three emulsion characteristics, which then may influence the tumor uptake and locoregional drug delivery.

From comparisons above, it is easy to understand that the crystallographical and chemical characteristics of Pll-nanoplex, which may satisfy all the nanometric features called for the combination of TAE-gene therapy, result in best cyto-tissue compatibility, safe procedure and excellent therapeutic efficiency in vitro and in vivo. Moreover, the application of lipiodol to nanoplex dramatically improved stability of nHAP emulsion, its stagnation in tumor target and favor its uptake by tumor cell. Indeed, thoroughly achievements and ideal transfection efficiency were not observed by using all the four systems in this study and the most frequently used polyethylenimine (PEI) in references [33, 34, 41, 47]. However, through comparing the four systems, a systemic requirement for nanometric features of materials in this new therapy is proposed and these guidelines may benefit the screening and identification for future systems. Many studies report the combination of Dosper liposome plus PEI 700 or 2000 as effective transfection synergism [33, 34, 41]. Recently, we has also managed to utilize branched PEI modified hydroxyapatite nanoparticles to transfer siRNA transfection of hepatoma cells in vitro [14]. Whether this combination can be applied in additional TAE-gene therapy in vivo is our future interest.

## Conclusion

We systematically apply and compare the usage of four different systems in vitro and in vivo. Though no better treatments is found than the former study [10], it is important to note that Pll-nHAP differs from unmodified nHAP, Ca-nHAP in several ways i.e., proper positive organic surface and smaller nano-sized diameter. Though the preliminary investigations in this study for the choice of synthetic material in hepatoma nano-TAE gene therapy is not adequate to draft defined guidelines concerning this issue, the practical experiences and mechanisms concluded could potentially be exploited to spur higher grade of evidence, particularly in vivo studies for TAE-gene therapy to HCC.

## Data Availability

The datasets generated during and/or analysed during the current study are available from the corresponding author on reasonable request.

## Abbreviations

Ca-nanoplex: Ca-nHAP-PEGFP-C2
HCC: Hepatocellular carcinoma
MFI: Mean fluorescence intensity
pDNA: Plasmid DNA
PEI: Polyethylenimine
Pll-nanoplex: Pll-nHAP-PEGFP-C2
Pll-nHAP: Hydroxyapatite nanoparticles
SEM: Scanning electron microscopes
TAE: Transcatheter arterial embolization
TE: Transfection efficiency
TEM: Transmission electron microscopes
TGW: Tumor growth rate
U-nanoplex: Unmodified nHAP-PEGFP-C2
wt-p53: Wild-type p53
